# Penile Cancer-Derived Cells Molecularly Characterized as Models to Guide Targeted Therapies

**DOI:** 10.3390/cells10040814

**Published:** 2021-04-06

**Authors:** Hellen Kuasne, Luisa Matos do Canto, Mads Malik Aagaard, Juan Jose Moyano Muñoz, Camille De Jamblinne, Fabio Albuquerque Marchi, Cristovam Scapulatempo-Neto, Eliney Ferreira Faria, Ademar Lopes, Sébastien Carréno, Silvia Regina Rogatto

**Affiliations:** 1 Department of Clinical Genetics, University Hospital of Southern Denmark-Vejle, Institute of Regional Health Research, University of Southern Denmark, 7100 Vejle, Denmark; hellen.kuasne@mcgill.ca (H.K.); luisa.matos.do.canto.alvim@rsyd.dk (L.M.d.C.); mads.jorgensen@rsyd.dk (M.M.A.); 2 International Research Center—A.C.Camargo Cancer Center, São Paulo 01508-010, Brazil; juan.moyano@unifesp.br (J.J.M.M.); fabio.marchi@accamargo.org.br (F.A.M.); 3 Institute for Research in Immunology and Cancer and Département de Pathologie et de Biologie Cellulaire, Université de Montréal, Montréal, QC H3C 3J7, Canada; camille.de.jamblinne.de.meux@umontreal.ca (C.D.J.); sebastien.carreno@umontreal.ca (S.C.); 4 Molecular Oncology Research Center, Barretos Cancer Hospital, Barretos 14784-400, Brazil; cristovam.neto.ext@dasa.com.br (C.S.-N.); elineyferreirafaria@yahoo.com.br (E.F.F.); 5 Diagnósticos da América—DASA, Barueri, São Paulo 06455-010, Brazil; 6 Uro-oncology and Robotic Surgery, Hospital Felicio Rocho, Belo Horizonte 30110-934, Brazil; 7 Pelvic Surgery Department, A.C.Camargo Cancer Center, São Paulo 01508-010, Brazil; ademar.lopes@accamargo.org.br

**Keywords:** penile cancer, cancer cell models, translatomic profile, genomic profile, protein expression, CAFs, EGFR inhibitors

## Abstract

Penile cancer (PeCa) is a common disease in poor and developing countries, showing high morbidity rates. Despite the recent progress in understanding the molecular events involved in PeCa, the lack of well-characterized in vitro models precludes new advances in anticancer drug development. Here we describe the establishment of five human primary penile cancer-derived cell cultures, including two epithelial and three cancer-associated fibroblast (CAF) cells. Using high-throughput genomic approaches, we found that the epithelial PeCa derived- cells recapitulate the molecular alterations of their primary tumors and present the same deregulated signaling pathways. The differentially expressed genes and proteins identified are components of key oncogenic pathways, including EGFR and PI3K/AKT/mTOR. We showed that epithelial PeCa derived cells presented a good response to cisplatin, a common therapeutic approach used in PeCa patients. The growth of a PeCa-derived cell overexpressing EGFR was inhibited by EGFR inhibitors (cetuximab, gefitinib, and erlotinib). We also identified CAF signature markers in three PeCa-derived cells with fibroblast-like morphology, indicating that those cells are suitable models for PeCa microenvironment studies. We thus demonstrate the utility of PeCa cell models to dissect mechanisms that promote penile carcinogenesis, which are useful models to evaluate therapeutic approaches for the disease.

## 1. Introduction

Penile cancer (PeCa) is an aggressive and mutilating disease that presents a high incidence in poor and developing countries [1,2]. According to two population-based cancer registry surveys, the survival of PeCa patients has not improved in Europe or the United States in the last three decades [3,4]. Although ongoing preclinical studies show promising results for more personalized therapy, the lack of improvement in survival is probably due to delayed diagnosis and lack of advances in curative standardized treatment options (reviewed in [5]).

Genetic and epigenetic alterations associated with PeCa development and progression have pointed out potential therapeutic targets (reviewed in [6,7]). In the last few years, we have reported molecular markers and deregulated pathways that are potentially helpful for discovering such targets in PeCa [8,9,10,11]. However, the scarcity of in vitro or animal models emerges as an obstacle that hampers the establishment of new and efficient therapeutic strategies for PeCa [12].

Cell cultures are valuable models for evaluating the molecular mechanisms underlying tumor initiation, progression, and drug response. Few cell lines derived from primary PeCa have been reported, and none of them are commercially available [13,14,15,16,17,18,19,20]. Two studies established and characterized cell lines derived from primary PeCa and corresponding lymph node metastasis [17,20]. We also reported a comprehensive characterization of a cell culture and xenograft derived from a verrucous tumor, which accounts for 2–8% of PeCa cases [10]. From 21 fresh PeCa samples, Chen et al. [18] established one cell line derived from a metastatic lymph node presenting a deleterious *TP53* mutation. This cell line was sensitive to cisplatin (commonly used in advanced PeCa) and epirubicin. The authors suggested that epirubicin was an effective chemotherapeutic agent, though it is not commonly used in PeCa treatment. Zhou et al. [19] established a panel of five PeCa cell lines sensitive to cisplatin. They reported that cell lines presenting EGFR DNA amplification and/or protein overexpression were resistant to anti-EGFR therapies. The authors presumed that *HRAS* and *PI3KCA* mutations might be related to resistance to anti-EGFR therapy. These studies have demonstrated that tumor-derived cell lines are good models for testing therapeutic agents and investigating drug resistance mechanisms, drug discovery, and targeted treatments.

Cells in the tumor microenvironment also play a crucial role in cancer progression and sensitivity to therapy. Studies in urological cancer types have drawn attention to the influence of stromal-epithelial interactions on tumor growth, invasion, and immune response [21,22,23]. Among stromal cells, cancer-associated fibroblasts (CAFs) modulate cancer metastasis through several mechanisms [24].

In the present study, we established and characterized five penile cancer-derived cells from cancer tissues (two epithelial and three CAFs). We evaluated the morphology of these cells and their ability to proliferate, migrate, and invade. Different -omics approaches were applied to characterize these derived PeCa cells molecularly. We showed that the tumor epithelial cells retained the genetic features of primary tissues. The response to cisplatin and EGFR inhibitors was also investigated. Overall, our results showed that these newly established cells could be used in pre-clinical assays to investigate drug response in PeCa.

## 2. Materials and Methods

Specimens derived from primary PeCa obtained after surgery from patients naïve of treatment were cultured under sterile conditions. The study was conducted following the Declaration of Helsinki and approved by the Human Research Ethics Committees of the A.C.Camargo Cancer Center (Protocol 1230/2009) and Barretos Cancer Hospital (Protocol 363-2010), São Paulo, Brazil. All subjects provided informed consent.

The clinical and pathological characteristics of the patients are described in Table 1. Five PeCa derived cells (cell 2 to cell 6) were successfully established. Cells 2, 3, and 6 were derived from mixed usual-sarcomatoid, verrucous, and basaloid PeCa subtypes, respectively. Cells 4 and 5 were derived from the usual subtype. The PeCa from patients 2, 5, and 6 showed a high clinical stage (III and IV). Only the PeCa from patient 2 was positive for the human papillomavirus (HPV16). A xenograft model derived from cell 3 was previously published by our group [10]. Translatomic and reverse-phase protein arrays (RPPA) for each tumor-derived cell (cells 2, 3, 4, 5, and 6) were compared with the foreskin cell line obtained from a healthy individual (cell 1), kindly donated by Dr. Silvya Stuchi Maria-Engler, Clinical Chemistry and Toxicology Department, University of São Paulo, SP, BR.

### 2.1. Establishment of Penile Cancer-Derived Cells

Histopathological analyses confirmed the presence of tumor cells in the samples used for culture. Five PeCa samples were dissociated and plated as previously described [10]. Briefly, minced tumor fragments were seeded in 25 cm^2^ culture flasks containing 3:1 KSFM (keratinocyte serum-free medium)–DMEM/F12 (Dulbecco’s modified Eagle medium/nutrient mixture F-12) (GIBCO, Carlsbad, CA, USA) added with 2.5% fetal bovine serum (FBS) (HYCLONE, Waltham, MA, USA), 30 µg/mL of bovine pituitary extract (BPE) (GIBCO, Carlsbad, CA, USA), 0.2 ng/mL of epithelial growth factor (EGF) (GIBCO, Carlsbad, CA, USA), antibiotics (100 IU/mL penicillin G and 100 mg/mL streptomycin) (Sigma-Aldrich, St. Louis, MO, USA), and 25 µg/mL of Fungizone^®^ Antimycotic (GIBCO, Carlsbad, CA, USA). After confluence, cells were treated with 0.05% trypsin/0.02% EDTA (Sigma-Aldrich, St. Louis, MO, USA) and replicated for at least 10 passages (P10).

### 2.2. Morphological Characterization and Functional Assays

The morphology of PeCa cells was evaluated by phase-contrast microscopy (Nikon TE2000, Amsterdam, Netherlands) and immunofluorescence by using Texas Red: actin/phalloidin (Thermo Fisher Scientific, Waltham, MA, USA), FITC (fluorescein isothiocyanate): tubulin (Thermo Fisher Scientific, Waltham, MA, USA), and DAPI (4′,6-diamidino-2-phenylindole): nucleus (Vector Laboratories, Burlingame, CA, USA).

The doubling time was calculated by seeding the cells at a density of 1.5 × 10^5^ cells in a 6 cm^2^ plate. Cell count was performed using Trypan blue (GIBCO, Carlsbad, CA, USA) dye exclusion in a Neubauer chamber every 24 h for eight days (the medium was replaced every three days). The doubling time was determined from the growth curves using the data from three independent experiments, each with three technical replicates.

Migration and invasion assays were conducted in transwell (Costar, Corning, New York, NY, USA) and Matrigel™-coated transwell plates (BD Biosciences, San Jose, CA, USA), respectively, as previously described [9]. Briefly, 5 × 10^5^ cells suspended in 100 µL of serum-free medium were added to the upper chamber of a transwell plate. The lower chamber contained 2.5% FBS (HYCLONE, Waltham, MA, USA), 30 µg/mL BPE (GIBCO, Carlsbad, CA, USA), and 0.2 ng/mL EGF (GIBCO, Carlsbad, CA, USA) supplemented medium, which acted as a chemoattractant, for 24 h at 37 °C. Cells incubated with serum-free medium only were used as controls. After fixation, the cells that migrated across the membrane were stained with hematoxylin-eosin. The number of cells that migrated and/or invaded were counted and scored from 0 to 4 (score 0: 0–5%, score 1: 5–25%, score 2: 25–50%, score 3: 50–75%, and score 4: 75–100%).

### 2.3. Molecular Profiling of Penile Cancer-Derived Cells

Genomic DNA and mRNA were isolated from all PeCa cells at passage 10. DNA was extracted using DNeasy Blood & Tissue Kit (Qiagen, Valencia, CA, USA). The human HPV genotyping was performed using the Linear Array HPV Genotyping Test (Roche Molecular Diagnostics, Branchburg, NJ, USA).

Copy number alterations (CNAs) and copy-neutral loss of heterozygosity (cnLOH) were assessed using the CytoScan HD platform (Thermo Fisher Scientific, Waltham, MA, USA). Data were analyzed with the Chromosome Analysis Suite 3.0 (ChAS) software (Thermo Fisher Scientific, Waltham, MA, USA, v.4.0), considering at least 25 markers for losses/mosaic losses, 50 markers for gains/mosaic gains, and cnLOHs with a minimum of 5 Mb, as previously described [25,26]. A distogram showing dissimilarity between samples (matched primary tumor and cell culture) was performed using the R package Rawcopy [27].

The mutational profiling of primary tumors and their derived cells was investigated by targeted next-generation sequencing (tNGS) of 105 cancer-related genes (SureSelectXT Custom Panel, Agilent, Santa Clara, CA, USA). The DNA libraries of all coding exons, intron–exon boundaries, and 3′ and 5′ UTR of the genes were prepared using SureSelectQXT Library Prep Kit (Agilent, Santa Clara, CA, USA). The DNA sequencing was carried out using 2 × 150-bp paired-end technology on the Next-Seq500 Illumina platform (Illumina, San Diego, CA, USA). All sequenced data were aligned to UCSC hg19. The initial analysis was performed as previously described [26]. Variants were filtered out based on their classification (benign—B or likely benign—LB, according to the ACMG—American College of Medical Genetics and Genomics or ClinVar), and frequency (>0.1 in the GnomAD v2.1.1—the Genome Aggregation Database [28], and/or in the ABRaOM—Online Archive of Brazilian Mutations [29] datasets). We excluded variants in homopolymer regions.

Actively translating ribosomes and their associated mRNAs were isolated, as described by Roffé et al. [30]. mRNA was extracted from polysome fractions using TRIzol reagent (Thermo Fisher Scientific, Waltham, MA, USA), according to the manufacturer’s recommendations. RNA quality and quantity assessment were conducted using an RNA 6000 Nano labchip (Bioanalyzer, Agilent, Santa Clara, CA, USA) and Nanodrop spectrophotometer (Thermo Fisher Scientific, Waltham, MA, USA), respectively. Translatomic profiling was investigated with the Clariom™ D Assay platform (Thermo Fisher Scientific, Waltham, MA, USA). Labeling, hybridization, and washing followed the manufacturer’s recommendations. Normalization, quality control, and analysis were assessed by the Transcriptome Analysis Console (TAC software Thermo Fisher Scientific, Waltham, MA, USA, v.4.0). The expression levels of PeCa-derived cells were compared to cell 1 (normal foreskin). Only probes with adjusted *p*-value < 0.001 and |fold change| > 4 were considered significant. Experiments were performed in duplicate. Expression levels of genes considered markers of CAFs or related to their activation were evaluated [31,32,33].

A high-throughput antibody-microarray-based technique (reverse-phase protein array—RPPA) was used to evaluate the protein expression profile in all PeCa-derived cells compared to cell 1. The RPPA experiments were performed in the RPPA core facility of MD Anderson Cancer Center, Houston, Texas, USA. Protein lysates were extracted according to the RPPA protocol (https://www.mdanderson.org/research/research-resources/core-facilities/functional-proteomics-rppa-core.html, accessed on 4 March 2021). Graphics representing RPPA results were build using the RPPApipe tool [34].

Gene set enrichment analysis was performed on the translatomic data using the single-sample gene set enrichment analysis (ssGSEA) tool (http://genepattern.broadinstitute.org, accessed on 6 April 2021). Annotated gene sets were obtained from the Reactome and oncogenic signature sub-collection of the Molecular Signature Database (MSigDB) (https://www.gsea-msigdb.org/gsea/msigdb/index.jsp, accessed on 6 April 2021). Ingenuity Pathway Analysis (IPA v01.12) (QIAGEN Inc, Germantown, MD, USA) was used to predict the top transcriptional regulators that are either activated or inhibited in PeCa-derived cells (upstream regulator analysis).

The gene expression levels of one study on penile cancer (GSE57955) publicly available [35] were assessed to identify the EGFR expression signatures (Reactome signaling by EGFR and KEEG ERBB signaling pathways). Heatmaps were generated using the Morpheus web tool (https://software.broadinstitute.org/morpheus/. accessed on 6 April 2021).

## 3. Results

### 3.1. Penile Cancer Derived-Cells Present Diverse Morphology, Proliferation, Migratory, and Invasive Characteristics

**Figure 1:**
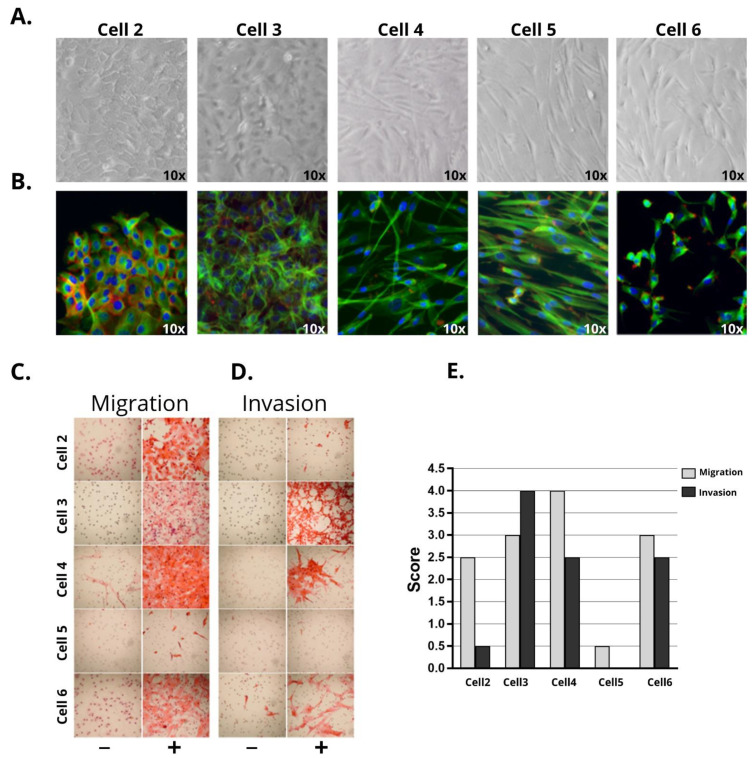
Morphological features, migration, and invasion characteristics of five penile cancer-derived cells. Cells were cultivated until passage P10. (**A**) Morphology of epithelial (cells 2 and 3) and fibroblast-like (cells 4, 5, and 6) cells by phase-contrast microscopy and (**B**) immunofluorescence (Texas Red: actin /phalloidin; FITC (fluorescein isothiocyanate): tubulin; and DAPI (4′,6-diamidino-2-phenylindole): nucleus) (10X magnification) (Nikon TE2000). Transwell migration and invasion assay (**C**,**D**) images and (**E**) quantification. The fields were evaluated in 4X magnification and were scored from 0 to 4 according to the percentage of cells that migrated and/or invaded (score 0: 0–5%, score 1: 5–25%, score 2: 25–50%, score 3: 50–75%, and score 4: 75–100%). Cells 2, 3, 4, and 6 presented high migratory capacity (scores 2.5 to 4), whereas cell 5 had a low score (score 0.5). Invasion capacity was observed in cells 3, 4, and 6. Chemotaxis was induced by adding 2.5% FBS (fetal bovine serum), BPE (bovine pituitary extract) (30ug/mL), and EGF (epithelial growth factor) (0.2 ng/mL) to the media, which is represented by (+). (−): control (media without chemo-attractants). Bars indicate the average of the scoring obtained in three independent experiments.

Two (cells 2 and 3) out of five PeCa-derived cells presented epithelial morphology (Figure 1A,B) whereas cells 4, 5, and 6 presented fibroblast-like morphology. Cells 2 and 3 showed positive expression of cytokeratin (Figure S1A) and epiplakin (VHL-PPK1) (Figure S2). Cell 3 presented both cytokeratin and vimentin expression (Figure S1A). Although cell 2 was derived from an HPV16 case, the positivity was lost after five passages in vitro.

Cells presenting the fibroblast-like morphology (Figure 1A,B) showed positive expression of vimentin in both immunocytochemistry and RPPA experiments, among other markers related to CAFs (Figures S1A and S2).

The doubling time was calculated for all cell cultures (Figure S1B). Cells 2, 3, 4, and 6 presented an average doubling time of 23.1 h, whereas cell 5 had a doubling time of 27.7 h (Figure S1B).

The ability of all cell lines to migrate and invade was evaluated using transwell assays. Cell 4 presented the highest average score of migration (score 4), followed by cells 3, 6 (score 3), and 2 (score 2.5), with cell 5 showing the lowest score (score 0.5) (Figure 1C,D). The invasive potential was high in cells 3, 4, and 6 (2 to 4 scores) and low in cell 2 (0 to 1 score), whereas cell 5 did not invade (0 score) (Figure 1D and Figure S1C). The ability to migrate and invade was not related to the morphology of the different cell lines.

### 3.2. Penile Cancer-Derived Cells Recapitulate the Molecular Profile of PeCa Primary Tissues

**Figure 2:**
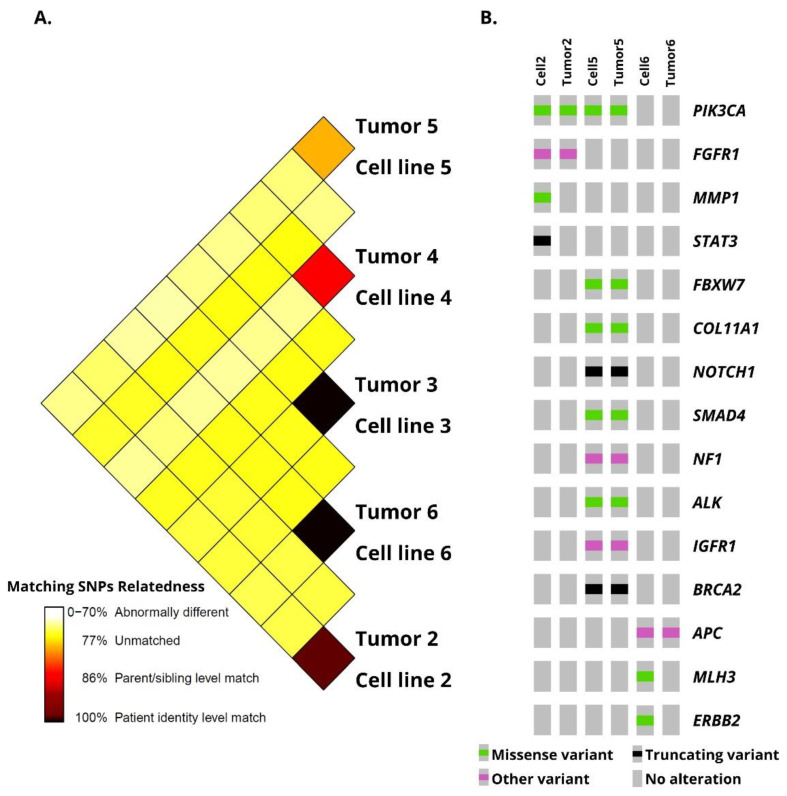
Genomic comparison of penile cancer (PeCa) samples and their derived cells (**A**) Sample identity distogram showing the degree of similarity among penile cancer-derived cells and their primary tumor tissue. (**B**) Variants identified by targeted next-generation sequencing in PeCa and derived PeCa cells. DNA from PeCa 3 and 4 were not available for this analysis. Oncoprint was generated by the cBioportal Oncoprinter tool (http://www.cbioportal.org/oncoprinter, accessed on 6 April 2021).

Genomic copy number alterations found in primary tumors were compared with their PeCa-derived cells (Cytoscan HD platform, Thermo Fisher Scientific, Waltham, MA, USA), revealing a high similarity level. The cells presented at least 80% matching single nucleotide polymorphisms with the primary tumor, confirming their parental origin (Figure 2A). In this analysis, cells 3 and 6 presented 100% genomic similarities with their matched primary tumors. A lower similarity was observed between the tumor and its derived cell 5 (~80%). Supplementary Table S1 and Figure S3 show the genomic alterations identified in primary tumors and their derived cells.

Overall, the primary tumors and their derived cells presented a lower number of chromosomal imbalances, with the highest number of altered regions in cell 3 (44) and the lowest in cell 4 (8). Cells with epithelial morphology shared a higher number of common alterations with the matched primary tissues. Primary tumor 2 and its derived cell 2 presented 24 chromosomal imbalances, including a homozygous loss of 9p21.3 (*CDKN2A* and *CDKN2B* genes). Cell 3 and its primary tumor 3 presented 28 genomic alterations in common (Table S1), including gains of 4q12 (*PDGFRA*), an amplification of 11p15.5 (*H19* and *IGF2* genes), and a loss of 6p25.3 (*DUSP22*). A loss of 8p, encompassing the *DLC1* gene, was identified only in cell 3.

Although CAFs shared common altered regions with their primary tumors, the alterations were detected in a lower frequency compared to epithelial cells. Three chromosomal imbalances (gains of 14q32.33 and losses of 11p15.4 and 5q23) were shared by tumor 4 and its derived cell 4. Primary tumor 5 and its derived cell 5 presented 13 CNAs, including gains of 3p12.1, 14q32.33, and 17q21.31 and losses of 5q23.1, 6p25.3, and 12p11.21. Cell 6 presented gains of 3q26.31 (*TNFSF10*), 14q32.33, 16p11.2, and 17q21.31, and losses of 5q35.3, 6p25.3, 8p11.22, 8p22, 12p13.33, and 19p12. Tumor 6 and its derived cell 6 presented the highest number of cnLOH (Figure S3).

We also detected similar genomic alterations in more than one derived tumor cell (Table S1). Among them, cells 2 and 3 shared a loss of 9p21.3 (*CDKN2A* and *CDKN2B*), all PeCa derived cells presented gains of 14q32.33, and cells 5 and 6 presented gains of 17q21.31 (*KANSL1*). A detailed description of the genomic alterations detected in our cells paired with their respective primary tumors is presented in Table S1 and Figure S3.

Targeted next-generation sequencing was performed in all PeCa-derived cells and three matching primary tumors (patients 2, 5, and 6) using a custom panel composed of 105 cancer-related genes. After filtering, we identified variants in all but one cell line (cell 3). A total of 18 single nucleotide variants - SNVs (nonsynonymous, stop gains, splice site, three and five prime untranslated regions) and two indels mapping to 17 genes were detected. The variants (missense, frameshift, and truncation) identified in the primary tumors and their matched derived cells are represented in Figure 2B and Table 2. All variants found in tumor 2 were also observed in cell 2 (two variants of *PIK3CA* and one of *FGFR1*). Tumor 5 and its derived cell 5 presented variants in nine different genes, among them *ALK*, *BRCA2*, *NOTCH1*, and *PIK3CA*. One variant in a CpG island, in the promoter region of the *APC* gene, was common to tumor 6 and its derived cell 6. Two variants were found exclusively in cell 2 and not in its tumor (*MMP1* and *STAT3*), whereas two variants were found only in the tumor from which cell 6 was derived (*ERBB2* and *MLH3*). The gene most frequently altered was *PIK3CA* (two variants in cell/tumor 2, one in cell/tumor 5). Table 2 summarizes the tNGS results found in the cells compared with the primary tumors.

### 3.3. Identification of Potential Therapeutic Targets in PeCa Cells Using Translatomic, Pathways, and Protein Analysis

**Figure 3:**
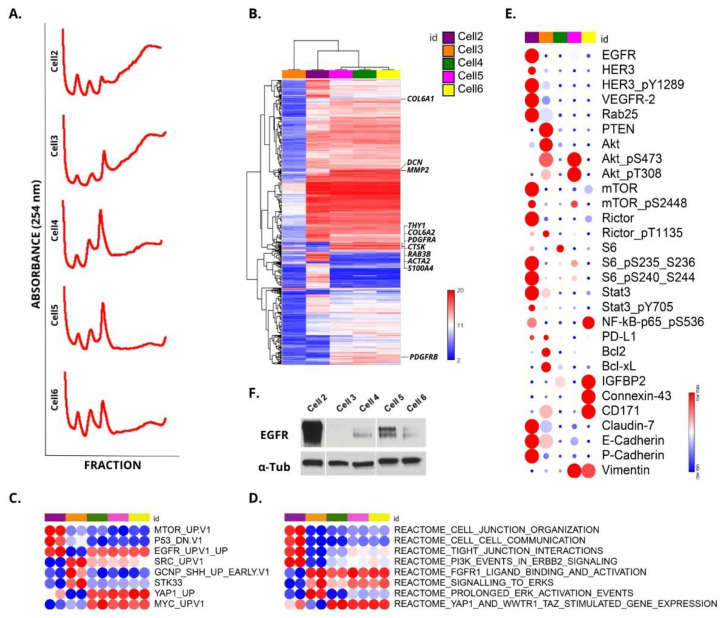
Molecular profile of penile cancer (PeCa)-derived cells. (**A**) Polysomal profiling of cells 2, 3, 4, 5, and 6. Polyribosome (polysome) fractionation was performed using a sucrose density gradient (5–50%). (**B**) Translatomic profiling (Clariom™ D Assay platform, Thermo Fisher Scientific, Waltham, MA, USA) was performed in duplicate for each derived PeCa cell culture and cell 1 (normal foreskin). A heatmap shows three clusters with probes differentially expressed compared to cell 1. The first cluster is composed of cell 3 replicates (tumor epithelial cells), cluster 2 is composed of cell 2 replicates (tumor epithelial cells), and the third one of cells 4, 5, and 6 (cancer-associated fibroblasts—CAFs). Blue: down-expressed probes, red: overexpressed probes. Genes indicated in the right of the heatmap are known markers of CAFs. (**C**,**D**) Gene set enrichment analysis was performed by ssGSEA (single-sample gene set enrichment analysis: https://www.genepattern.org/modules/docs/ssGSEAProjection/4, accessed on 6 April 2021). Oncogenic signature and Reactome sub-collection came from the Molecular Signature Database (MySigDB) (https://www.gsea-msigdb.org/gsea/msigdb/index.jsp, accessed on 6 April 2021). Red: highest score in the ssGSEA, blue: lowest score. UP: up-regulated genes; DN: downregulated genes; V1: version 1. (**E**) Heatmap of reverse phase protein array (RPPA) representing proteins or phosphorylated isotypes in PeCa cells. The protein or phosphorylated isotype expression levels in tumor cells was compared to cell 1 (fold change—FC). The FC levels of each biomarker of the five penile cancer-derived cells are indicated in colors (red for the highest and blue for the lowest). (**F**) Western blot showing EGFR overexpression in cell 2 compared to the other PeCa cells.

To evaluate the actively translated mRNAs, we performed a polysome profiling analysis using a sucrose gradient, which involves the separation of mRNAs into fractions according to the number of bound ribosomes. The mRNA profile from the polysomal fraction of each cell was investigated using the Clariom™ D Assay platform (ThermoFisher, USA) (Figure 3A,B). A heatmap containing the top 250 differentially expressed genes (DEG) showed three clusters. The first was composed of cell 3 (epithelial morphology), the second cluster of cell 2 (epithelial morphology), and the third cluster of cells 4, 5, and 6 (fibroblast-like morphology—CAFs). Penile cancer-derived cells 4, 5, and 6 presented overexpression of markers related to a CAF signature (*MMP2, REAB3B, COL6A1, COL6A2, CTSK, THY1, PDGFRA, DCN,* and fibroblast activation *ACTA2*) [31,32] (Figure 3B). The fibroblast activation protein α gene (*FAP*) did not show increased expression. The top 15 DEG of cells 2 to 6 compared with cell 1 (normal foreskin) and the number of DEGs for each comparison are shown in Table 3.

A single-sample gene set enrichment analysis (ssGSEA) was performed, and the main pathways identified as dysregulated in the PeCa cells are depicted in Figure 3C,D. Using oncogenic signatures (Figure 3C) and the Reactome (Figure 3D) sub-collection of the MSigDB, we identified that EGFR, PI3K, and mTOR pathways were dysregulated in cell 2. Cell 2 also presented a high score for several cell junction signatures (Figure 3D). Cell 3 expressed genes related to the regulation of apoptosis (*BCL2L1* and *BCL2*) and pathways related to SRC and ERK activation. Cells 4, 5, and 6 presented pathways related to the CAF phenotype, including YAP and MYC signatures (Figure 3C,D).

We also evaluated the protein profile of 304 pre-selected antibodies involved in pathways potentially dysregulated in cancer (Figure S2). Figure 3E shows the main altered proteins or phosphorylated isotypes in PeCa cells. Potential therapeutic targets were identified among the dysregulated proteins identified in the PeCa cells. Cell 2 presented increased expression of EGFR, HER3, and VEGFR2, which are described as targets for therapy and tested in clinical trials (NCT01728233—dacomitinib). Increased EGFR expression was observed in cell 2 (Figure 3F). In addition, several proteins downstream to the EGFR receptor were dysregulated (Figure 3E). Cell 2 also presented a decreased expression of p16INK4A, which correlates with the genomic deletion of the *CDKN2A* gene detected by CNA analysis (Figure S3A).

High levels of phosphorylated AKT were observed in cells 3 and 5. The epithelial cells marker, epiplakin (VHL-PPK1), was highly expressed in cells 2 and 3. A weak expression of epiplakin was observed in cell 4, whereas no expression of this marker was found in cells 5 or 6. Vimentin, a marker of mesenchymal cells, was expressed in cells 5 and 6 (Figure 3E and Figure S2).

The expression levels of PI3K/AKT/mTOR, EGFR, ERBB2, TP53, and CDKN2A proteins found in our cells were previously reported in PeCa (Table 4). In addition to EGFR and CDKN2A described above, we also detected alterations in targetable tyrosine kinase receptors, including ERBB2 and ERBB3, and several proteins downstream to this pathway, such as AKT, S6, and mTOR (Table 4, Figure 3E).

### 3.4. Identification of Potential Therapeutic Targets for PeCa and Chemo-Sensitivity Assays

**Figure 4:**
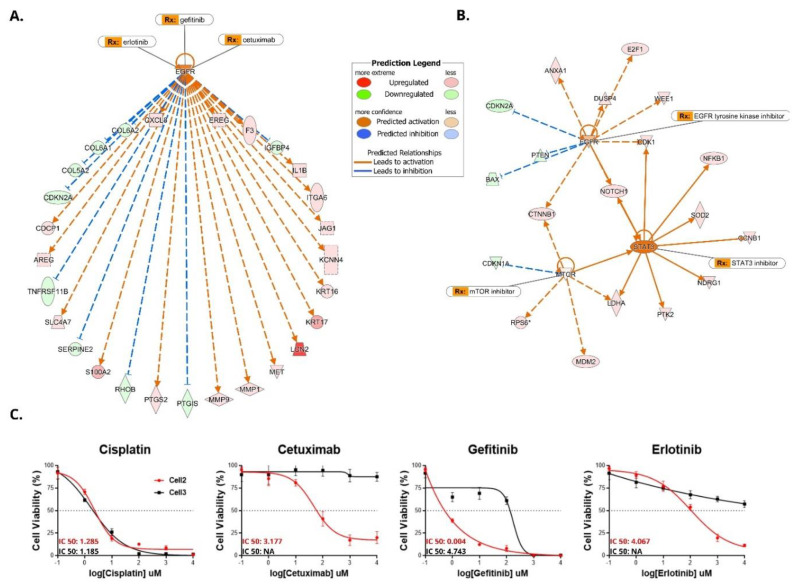
Enrichment analysis of direct and indirect mRNA interactions and proteins dysregulated in penile cancer (PeCa) cells show EGFR as a core molecule. Sensitivity assays to EGFR inhibitors are depicted for cells 2 and 3. Upstream regulator analysis (IPA, Ingenuity Pathway Analysis) indicates direct (solid lines) and indirect (dashed lines) interaction of mRNA (**A**) and proteins, which is exemplified in PeCa-derived cell 2. (**B**) EGFR is a core molecule in both mRNA and protein analyses. (**C**) Chemosensitivity assays were performed in cell 2 and cell 3 for the identification of IC50 and dose-response curves. Cells were treated with cisplatin and EGFR inhibitors (cetuximab, gefitinib, and erlotinib). Cell 2 and cell 3 were sensitive to cisplatin. Only cell 2 (EGFR overexpression) was sensitive to EGFR inhibitors.

We performed chemo-sensitivity assays in epithelial PeCa cells 2 and 3. Using the Ingenuity Pathway Analysis software (Qiagen, Valencia, CA, USA), we identified potential drugs that target the differentially expressed kinases and proteins that showed higher expression (Table 5). The upstream regulator analysis of cell 2 revealed several genes predicted to be regulated by EGFR (Figure 4A,B). EGFR was a core molecule in both mRNA (Figure 4A) and protein analysis in cell 2 (Figure 4B).

Based on the evidence that EGFR was dysregulated in cell 2 at mRNA and protein (RPPA and Western blot) levels (Figure 3E,F), we tested the chemo-sensitivity of this cell using anti-EGFR inhibitors (cetuximab, gefitinib, and erlotinib). Cell 3 was used as a negative control since it did not show EGFR overexpression. We also tested cell viability in response to cisplatin (commonly used in the treatment of advanced PeCa) in cells 2 and 3. The IC50 and dose-response curve results showed that these cell lines were sensitive to cisplatin. However, only cell 2 (EGFR overexpression) was sensitive to anti-EGFR drugs (Figure 4C).

**Figure 5:**
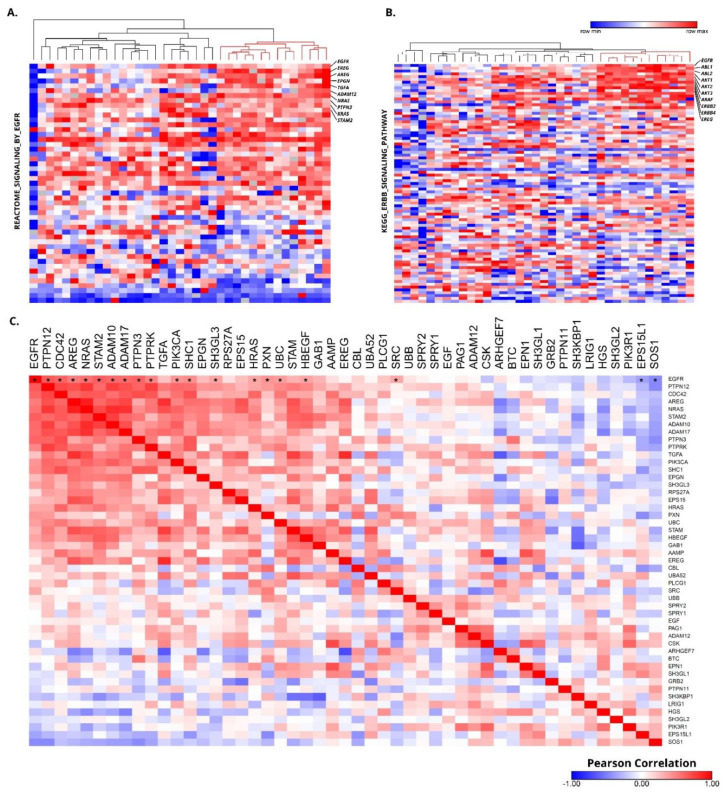
Approximately 30% of penile cancer overexpresses genes related to the EGFR signaling pathway. Heatmap illustrating the Reactome signaling by EGFR (50 genes) (**A**) and KEGG ERBB signaling pathway (87 genes) (**B**) signature of 36 PeCa samples (GSE57955). Rows represent normalized mRNA expression for a single gene (Agilent microarray, Agilent, Santa Clara, CA, USA), and each column represents one of the 36 previously analyzed PeCa samples. All tumor samples were normalized with a pool of normal samples, and the values were ranked by their expression levels (blue: down-expressed genes, red: overexpressed genes). The red cluster (**A**,**B**) presents overexpression of several genes belonging to the EGFR signature. (**C**) Heatmap representing a matrix of the Pearson correlation of genes from the Reactome signaling by EGFR in the 36 PeCa samples (GSE57955). *EGFR* (first column) was positively correlated with several genes of the PIK3 and MAPK pathways. A significant P-value of Pearson correlation is represented by (*) only for the *EGFR* gene (first line).

Having shown that EGFR inhibition can block the proliferation of PeCa cells in vitro, we evaluated whether PeCa primary tissues overexpress the *EGFR* gene. Samples that presented overexpression of the EGFR signature most likely have activation of the EGFR pathway. We accessed EGFR mRNA-related signatures (MySigDB) in a set of 36 primary PeCa tissues previously published by our group (GSE57955) [35]. We identified a cluster of samples (~30% of the cases) showing *EGFR* mRNA signature overexpression (Figure 5A,B). Several genes from the *EGFR* signature (downstream to *EGFR*) were positively correlated with *EGFR* expression (Figure 5C).

## 4. Discussion

An in vitro culture of tumor cells is a valuable model for drug development and preclinical drug testing in oncology. At least in part, these cells maintained the molecular alterations of the parental tumor and thus can be used in functional studies [56,57]. However, establishing cancer cell lines from fresh tumor tissues represents a technical challenge [58]. These models also present certain limitations (such as the lack of tumor heterogeneity, the absence of components of the tumor microenvironment, and genotypic and phenotypic drift during culture) that must be taken into consideration [59].

The development of in vitro models of PeCa is hampered by the low incidence of this tumor type. In this study, we derived PeCa cells from fresh penile primary tumors. Two of the established PeCa-derived cells presented a typical polygonal epithelial cell morphology (cells 2 and 3), and three presented fibroblast-like morphology (cells 4, 5, and 6). These CAFs are components of the tumor microenvironment playing crucial roles in tumor progression and treatment response [60,61]. Interesting approaches were developed by Di Donato et al [23]., where the prostate tumor growth was stimulated by androgen in co-cultures using CAFs derived from patients with positive AR (androgen receptor) and cell lines that were either AR positive or negative [23]. Bladder cancer progression, migration, and invasion were also shown to be triggered by factors secreted by CAFs [22]. In both cases, inhibiting CAF signaling sufficed to decrease tumor aggressiveness [22,23]. Although several studies indicated the influence of the microenvironment in response to chemotherapy, we selected only cells showing epithelial features (cells 2 and 3) to evaluate the treatment response since the selected drugs target the tumor cells and not the microenvironment.

The primary tumor from patient 2 was positive for high-risk HPV, whereas the derived cell 2 was HPV negative. Attempts to reproduce HPV replication in standard cell culture have been unsuccessful, mostly because the replication process is linked to the differentiation of keratinocytes, and it is challenging to recreate the stratified structure of the epithelium in vitro [12,62].

The primary tumor that generated cell 2 was a mixed tumor (usual with few sarcomatoid differentiation areas). Sarcomatoid PeCas are rare and aggressive tumors (1–2% of PeCa) associated with metastasis and poor prognosis [63,64]. Using phase-contrast microscopy, we exclusively detected epithelial cells without spindle cells, expected in the sarcomatoid component (Figure 1A,B). Indeed, microarray and RPPA analysis showed the expression of several epithelial markers in cell 2, including *KRT5, KRT17, CDH1, DSG3* (Table 3), and epiplakin (VHL-PPK1) (Figure S2). These results suggest that only the usual component of this mixed PeCa was selected in vitro.

Very few studies have evaluated the mRNA expression profile of PeCa cell lines [18,19]. To our knowledge, we were the first to evaluate the expression levels of ribosome-associated mRNAs (translatomic analysis) in PeCa-derived cells. Since these mRNAs are associated with several ribosomes, they are likely actively translated [65]. Cell 2 was the only PeCa-derived cell that presented high EGFR overexpression at gene and protein levels. Gene set enrichment analysis (Figure 3C,D) and upstream regulator analysis (Figure 4A,B) also showed that several genes downstream to the EGFR pathway and EGFR interactors were dysregulated. Silva Amancio et al. [42] reported increased EGFR protein expression (3+: 67 of 139 cases) and its association with cancer recurrence and perineural invasion. Chaux et al. [66] found EGFR overexpression in 44% of the PeCas evaluated. These studies suggested that targeting the EGFR pathway would be an effective therapeutic strategy to treat a subset of cases. In contrast, Zhou et al. [19] identified EGFR protein overexpression (Western blot) in four of five PeCa cell lines and resistance to erlotinib and afatinib. Only one of these cell lines showed a minor sensitivity to cetuximab. The authors suggested that *HRAS* and *PI3KCA* alterations might be related to anti-EGFR therapy resistance. A chemo-sensitivity assay with a panel of drugs targeting EGFR (cetuximab, gefitinib, and erlotinib) showed that cell 2 was sensitive to anti-EGFR drugs (Figure 4C).

Anti-EGFR agents have been used to treat PeCa patients, mainly as a salvage treatment after first-line chemotherapy failure [67,68,69]. Di Lorenzo et al. [67] reported that 50% of 28 patients (24 treated with cetuximab) were sensitive to anti-EGFR monoclonal antibodies [67]. PeCa patients treated with nimotuzumab after chemotherapy failure showed clinical response or stable disease [69]. Necchi et al. [70] reported that dacomitinib (pan-HER inhibitor) was active and well-tolerated in patients with advanced PeCa and may represent an option when combined chemotherapy cannot be administered. The evaluation of downstream effectors of EGFR signaling and how these mutations can affect therapy response over time should be taken into account. Cell 2 presented a good initial response against EGFR inhibitors, but the occurrence of the two *PIK3CA* pathogenic variants could be translated into the later acquisition of resistance to treatment, as described in metastatic colorectal cancer [71]. However, it was not possible to confirm this assumption because the patient was only treated by surgery and lost to follow-up. The effect of *PIK3CA* mutation and resistance to EGFR inhibitors in PeCa should be better investigated. Other receptors such as ERRB3, phospho-ERBB3, and VEGFR2 were also overexpressed in this cell line and represent potential therapeutic opportunities. 

Several potentially targetable dysregulated oncogenic pathways have been described in PeCa [9,38,72,73,74], including PI3K/Akt/mTOR, EGFR, and ERRB2, among others. Recently, clinical trials testing immunotherapy in PeCa patients have shown promising results [75]. Positive expression of PD-L1 was found in 48–60% of cases [75], indicating that a significant proportion of these patients do not respond to immune-checkpoint inhibitors. Genetic studies have shown that the PI3K/Akt/mTOR pathway is significantly altered in PeCa cases [9,38,73,74]. Here, cells 2 and 5 presented alterations involving the PI3K/Akt/mTOR signaling pathway axis. We identified several mechanisms involved in the dysregulation of this pathway in PeCa cells, including *PIK3CA* mutation and increased expression levels of mTOR, RICTOR, phosphorylated S6 (cell 2), and phosphorylated AKT (cell 5), among others. The PI3K/AKT/mTOR signaling pathway has been associated with cell proliferation, migration, metabolism, and survival [76]. This pathway was dysregulated in cell 5, suggesting that the microenvironment cells can also be a potential target for cancer therapy [77].

Previously we described a similar genomic profile of cell 3 at passages 1, 5, and 10, thus showing genomic stability after cell culture [10]. Here, we used translatomic and protein expression analyses to better characterize this cell line. The enrichment analysis using DEGs revealed increased expression levels of Hedgehog pathway genes. Hedgehog signaling pathway inhibitors have been developed for cancer treatment [78]. Cell 3 also presented the dysregulation of other targetable pathways such as SRC and ERK (Figure 3C,D), widely investigated in several tumor types. This PeCa-derived cell presented a complex pattern of gene alteration that affected several oncogenic pathways. Thus, combined therapies targeting one or more dysregulated pathways are promising alternatives to treat PeCa, and clinical trials should be encouraged.

We showed that cell 2 and cell 3 were sensitive to cisplatin, but only cell 2 (EGFR overexpression) was sensitive to anti-EGFR drugs (Figure 4C). As expected, cell 3 did not respond to EGFR inhibitors. The EGFR status is a crucial step to be investigated before treating patients with EGFR inhibitors. The in-depth molecular characterization used in our PeCa-derived cells can assist the selection of drugs to be tested in representative models of PeCa.

The genomic analysis performed in five penile cancer-derived cells revealed a high level of similarities with the primary tumors. The most relevant differentially expressed genes and proteins are components of critical oncogenic pathways supporting previous studies published in the literature. We identified proteins with the potential to be targeted in PeCa patients, including EGFR, VEGFR2, PIK3/AKT/mTOR, MAPK, and SRC. Considering that our PeCa-derived cells closely recapitulated the molecular features of their primary tumors and presented the same dysregulated pathways, they are excellent models to be used in preclinical tests. Moreover, the cancer-associated fibroblasts can be used for microenvironment-related studies.

## Figures and Tables

**Table 1:** Clinical and pathological characteristics of penile cancer patients.

Patient/ Cell Line	Age (Years)	Histological Subtype	Surgery Type	HPV	TNM	Perineural Invasion	Clinical Stage	Follow-up (Months)
2/Cell 2	85	Usual + Sarcomatoid	Partial penectomy	HPV16	T3N0M0	Yes	III	120
3/Cell 3	71	Verrucous	Partial penectomy	Negative	T1N0M0	No	I	138
4/Cell 4	43	Usual	Partial penectomy	Negative	T1N0M0	Yes	I	102
5/Cell 5	57	Usual	Total penectomy	Negative	T4N0M1	Yes	IV	30
6/Cell 6	70	Basaloid	Partial penectomy	Negative	T2N1M2	No	IV	18

HPV—human papillomavirus; TNM—TNM Staging System (T: Tumor size, N: regional lymph nodes, M: Distant metastasis).

**Table 2:** Variants identified by targeted next-generation sequencing in the penile cancer-derived cell cultures.

ID	Gene	Classification	Chr: Location	Type of Alteration	dbSNP	Transcript	Base Change
Cell 2/tumor 2	*PIK3CA*	P	3:17,8936,082	Missense	rs121913273	NM_006218.4	c.1624G > A
Cell 2/tumor 2	*PIK3CA*	P	3:178,936,093	Missense	rs121913275	NM_006218.4	c.1635G > C
Cell 2/tumor 2	*FGFR1*	VUS	8:38,270,403	Other	rs1364534792	NM_023110.3	c.*743dupA
Cell 2	*MMP1*	VUS	11:102,667,445	Missense		NM_002421.4	c.575G > C
Cell 2	*STAT3*	P	17:40,486,045	LoF		NM_139276.2	c.820C > T
Cell 3	*-*						
Cell 4	*RAD50*	VUS	5:131,953,850	Missense	rs143189763	NM_005732.4	c.3253A > G
Cell 4	*MMP1*	VUS	11:102,668,717	LoF	rs139018071	NM_002421.4	c.105+2T > C
Cell 4	*FLT3*	LP	13:28,589,804	Missense	rs903856095	NM_004119.3	c.2576G > A
Cell 5/tumor 5	*COL11A1*	LP	1:103,380,339	Missense		NM_001854.4	c.3845G > T
Cell 5/tumor 5	*ALK*	VUS	2:29,455,260	Missense		NM_004304.5	c.2542G > A
Cell 5/tumor 5	*PIK3CA*	P	3:178,952,085	Missense	rs121913279	NM_006218.4	c.3140A > G
Cell 5/tumor 5	*FBXW7*	LP	4:153,247,367	Missense	rs747241612	NM_001349798.2	c.1435C > G
Cell 5/tumor 5	*NOTCH1*	P	9:139,413,064	LoF		NM_017617.5	c.1078G > T
Cell 5/tumor 5	*BRCA2*	P	13:32,968,951	LoF	rs80359212	NM_000059.3	c.9382C > T
Cell 5/tumor 5	*IGF1R*	VUS	15:99507206	Other		NM_000875.5	c.*6535T > G
Cell 5/tumor 5	*NF1*	VUS	17:29,702,854	Other	rs909909591	NM_001042492.3	c.*1683_*1685delGAA
Cell 5/tumor 5	*SMAD4*	LP	18:48,575,116	Missense		NM_005359.6	c.310C > T
Cell 6/tumor 6	Near *APC*	VUS	5:112,043,188	upstream transcript variant	rs1554060178	NC_000005.10	112707490:C:G
Tumor 6	*MLH3*	VUS	14:75,483,802	Missense		NM_001040108.1	c.4345C > T
Tumor 6	*ERBB2*	LP	17:37,866,662	Missense		NM_004448.3	c.829G > T

Chr: chromosome. c. represents the coding sequence position. LoF: Loss of function. P: Pathogenic. LP: Likely pathogenic. VUS: variant of uncertain significance. dbSNP: The Single Nucleotide Polymorphism Database

**Table 3:** Top 15 differentially expressed genes in penile cancer cells compared with the normal reference cell (cell 1). Translatomic profiling was performed using the microarray platform Clariom™ D Assay platform (Thermo Fisher Scientific, Waltham, MA, USA) after polysomal mRNA enrichment. The number within brackets represents differentially expressed genes after enrichment analysis, indicating genes more likely to be translated into proteins.

Cell 2 (564)		Cell 3 (1199)		Cell 4 (262)		Cell 5 (205)		Cell 6 (163)	
**Top 15 Overexpressed Genes**									
***Gene***	***FC***	***Gene***	***FC***	***Gene***	***FC***	***Gene***	***FC***	***Gene***	***FC***
*S100A8*	54897.5	*OSR1*	1865.57	*SFRP2*	1936.2	*IL24*	305.3	*TMEM176B*	2376.2
*COL17A1*	35757.4	*FOXG1*	1259.5	*TRPA1*	964.7	*APOD*	101.4	*TMEM176A*	1686.2
*KRT5*	25856.7	*PRKAR2B*	619.61	*LINC01436*	458.9	*SFRP2*	97.6	*APOD*	935.5
*S100A9*	25339.7	*HOXD8*	333.72	*TMEM176B*	288.4	*STMN2*	83.6	*SLC14A1*	873.7
*LCN2*	23704.2	*LGALSL*	172.59	*TMEM176A*	252.5	*EGR1*	79.7	*XGY2*	446.0
*CDH1*	18954.7	*PORCN*	119.5	*XGY2*	247.8	*XG*	66.9	*XG*	374.6
*LAMC2*	17269.9	*NRN1*	86.36	*SAT1*	113.8	*SNORD50A*	59.6	*CCND2*	343.2
*MAL2*	15941.8	*TBX15*	74.35	*XG*	100.1	*ZBTB16*	51.1	*FAM105A*	213.8
*GJB2*	14969.1	*PRPF39*	63.2	*IL33*	98.9	*TNFRSF21*	44.0	*SAA1*	163.9
*FXYD3*	13924.8	*MSI2*	41.57	*APOD*	98.2	*DUXAP10*	40.9	*STMN2*	160.7
*KRT17*	11118.8	*SLC44A1*	40.69	*OSR1*	88.5	*USP53*	37.3	*AQP1*	158.7
*DSG3*	10259.7	*STX6*	36.88	*PDGFRL*	72.3	*LINC01296*	31.6	*IL13RA2*	155.7
*FGFBP1*	10054.2	*CUL3*	34.26	*CCND2*	65.9	*RGCC*	25.6	*DUSP6*	147.2
*PI3*	10052.7	*S100A7*	32.87	*CLU*	62.3	*SFRP1*	22.5	*FMO3*	138.3
*S100A2*	9269.6	*RTCB*	29.47	*FGF7*	58.3	*EIF3A*	21.5	*ITGA8*	138.2
**Top 15 Down-Expressed Genes**									
***Gene***	***FC***	***Gene***	***FC***	***Gene***	***FC***	***Gene***	***FC***	***Gene***	***FC***
*BGN*	–15958.7	*CCL2*	–24130.3	*GFRA1*	–1175.8	*GFRA1*	–1156.6	*GFRA1*	–738.2
*GREM1*	–14798.9	*CLDN11*	–9744.1	*ADAM12*	–1155.9	*ADAM12*	–615.7	*CPA3*	–522.3
*UCHL1*	–13551.2	*UCHL1*	–9705.2	*IGFBP3*	–556.8	*POSTN*	–585.0	*POSTN*	–468.1
*MGST1*	–13191.8	*LUM*	–9606.2	*PSG5*	–416.9	*CPA3*	–422.7	*CXCL12*	–169.9
*THY1*	–11417.6	*SULF1*	–7813.0	*CPA3*	–411.1	*UCP2*	–309.4	*PLPP4*	–147.7
*MXRA8*	–8181.8	*HIST1H2BM*	–7623.5	*PLPP4*	–378.8	*UCHL1*	–255.8	*PSG2*	–119.4
*LOXL1*	–8004.0	*MXRA8*	–6464.9	*CNN1*	–365.3	*ACKR3*	–209.7	*PSG1*	–106.9
*ENG*	–7918.5	*PSG5*	–6399.8	*CLDN11*	–275.9	*CNN1*	–189.1	*HIST1H3F*	–96.9
*FBLN5*	–6160.9	*MGST1*	–6082.7	*PLPPR4*	–245.8	*COL1A1*	–133.3	*BEX1*	–95.9
*MFAP4*	–3745.0	*MRPL20*	–4740.9	*UCP2*	–243.7	*HIST1H3F*	–114.1	*PSG8*	–66.6
*ADGRA2*	–3508.0	*FARP1*	–4690.7	*LBH*	–220.6	*KRT7*	–79.1	*SHOX*	–62.4
*COL1A2*	–2748.2	*THY1*	–4498.7	*PSG8*	–167.9	*MYBL2*	–73.4	*UCP2*	–55.3
*GFRA1*	–2705.4	*TGFBR1*	–4386.3	*SLC7A5*	–162.6	*LYPD6B*	–63.9	*DAPK1*	–49.0
*F2RL2*	–2520.2	*HIST2H4B*	–4137.9	*PSG11*	–160.0	*SEL1L3*	–53.0	*KCND3*	–45.9
*MYADM*	–2340.8	*CSRP1*	–4129.1	*MEST*	–138.6	*KCND3*	–44.6	*COLEC12*	–45.2

FC: fold change.

**Table 4:** Summary of the studies in penile cancer that evaluated the expression (immunohistochemistry or immunofluorescence) of the same proteins presented in our RPPA (reverse-phase protein arrays) analysis.

Protein	Number of Cases	Expression	Reference	Cell				
				2	3	4	5	6
Akt1	148	↑	Stankiewicz et al., 2011 [36]	↓	↑	-	↑	-
pAkt	112	↑	Chaux et al., 2014 [37]	-	↑	-	↑	↓
pAkt	148	↑	Stankiewicz et al., 2011 [36]	-	↑	-	↑	↓
pAkt	57	↑	Azizi et al., 2019 [38]	-	↑	-	↑	↓
ARID1A	112	↑	Faraj et al., 2015 [39]	↑	↑	↓	↑	↑
c-MET	92	↑	Gunia et al., 2013 [40]	-	-	-	-	-
c-MYC	141	↓	Arya et al., 2015 [41]	-	-	-	-	↓
cyclinD1	141	↓	Arya et al., 2015 [41]	-	-	-	-	-
EGFR	139	↑	Silva Amancio et al., 2017 [42]	↑	-	-	↑	-
EGFR	52	↑	Dorff et al., 2016 [43]	↑	-	-	↑	-
EI2F	13	↑	Fenner et al., 2018 [20]	↑	↑	-	↑	-
eIF4E	67	↑	Ferrandiz-Pulido et al., 2013 [44]	↑	↑	-	-	↓
peIF4E	67	↑	Ferrandiz-Pulido et al., 2013 [44]	-	-	-	-	-
HER2	148	↑	Stankiewicz et al., 2011 [36]	↑	↑	-	-	-
HER3	148	↑	Stankiewicz et al., 2011 [36]	↑	↑	-	-	-
HER4	148	↑	Stankiewicz et al., 2011 [36]	-	-	-	-	-
p16	58	↑	Steinestel et al., 2015 [45]	↓	↓	-	↓	↓
p16	202	↑	Cubilla et al., 2011 [46]	↓	↓	-	↓	↓
p16	119	↑	Tang et al., 2015 [47]	↓	↓	-	↓	↓
p16	123	↑	Mannweiler et al., 2013 [48]	↓	↓	-	↓	↓
p4E-BP1	67	↑	Ferrandiz-Pulido et al., 2013 [44]	↑	↑	-	-	-
p53	123	↑	Mannweiler et al., 2013 [48]	-	-	-	-	-
p53	110	↑	Gunia et al., 2013 [49]	-	-	-	-	-
p53	297	↑	Rocha et al., 2012 [50]	-	-	-	-	-
PDL1	116	↑	Deng et al., 2017 [51]	-	↑	-	-	-
PDL1	37	↑	Udager et al., 2016 [52]	-	↑	-	-	-
PDL1	52	↑	Cocks et al., 2017 [53]	-	↑	-	-	-
PDL1	213	↑	Ottenhof et al., 2017 [54]	-	↑	-	-	-
pmTOR	67	↑	Ferrandiz-Pulido et al., 2013 [44]	↑	-	-	-	↑
pmTOR	112	↑	Chaux et al., 2014 [37]	↑	-	-	-	↑
PTEN	112	↓	Chaux et al., 2014 [37]	-	-	-	-	-
PTEN	148	↓	Stankiewicz et al., 2011 [36]	↓	↑	-	↓	↓
PTEN	57	↓	Azizi et al., 2019 [38]	↓	↑	-	↓	↓
pS6	57	↑	Azizi et al., 2019 [38]	↑	-	-	↑	-
SOD2	125	↑	Termini et al., 2015 [55]	↑	↑	↑	↑	-

↑: increased expression; ↓: decreased expression; -: no change in expression levels.

**Table 5:** Drugs that target receptor tyrosine kinase genes and proteins. The tyrosine kinases showed high expression levels (fold change > 2) in penile cancer-derived cells 2 and 3. Data were generated using the Ingenuity Pathway Analysis software (Qiagen, Valencia, CA, USA).

Cell Line	Gene Symbol ^a^	mRNA FC	Location	Drug(s) ^b^
**mRNA**				
Cell 2	*FRK*	144.007	Nucleus	Regorafenib
Cell 2	*ERBB3 ^c^*	121.938	Plasma membrane	Afatinib/cetuximab/osimertinib/erlotinib
Cell 2	*MST1R*	54.569	Plasma membrane	Crizotinib/erlotinib/gefitinib/pazopanib
Cell 2	*PRKDC*	43.713	Nucleus	CC-115/MSC2490484A/panulisib
Cell 2	*EGFR* ^c^	38.586	Plasma membrane	Gefitinib/erlotinib/afatinib/cetuximab
Cell 2	*MET*	30.91	Plasma membrane	Crizotinib/cabozantinib/ABT-700/altiratinib
Cell 3	*SRC*	28.443	Cytoplasm	Dasatinib/bosutinib/blinatumomab/AZD0424
Cell 2	*DDR1*	24.59	Plasma membrane	Blinatumomab/dacomitinib/dasatinib/imatinib
**RPPA**				
Cell 2	*KDR*	4.729	Plasma membrane	5-azacytidine/sorafenib/sAEE788/apatinib
Cell 2	*RPS6KA1*	3.775	Cytoplasm	PMD-026
Cell 2	*MAP2K1*	3.493	Cytoplasm	ARRY-424704/AS703988/binimetinib
Cell 2	*MTOR*	2.925	Nucleus	ABI-009/apitolisib/sirolimus/tacrolimus/everolimus
Cell 2	*SRC*	2.792	Cytoplasm	AZD0424/dasatinib/bosutinib/ponatinib
Cell 3	*CDK1*	2.637	Nucleus	Alvocidib/dinaciclib/milciclib/riviciclib
Cell 2	*MKNK1*	2.62	Cytoplasm	BAY1143269/dacomitinib/ETC-1907206/tomivosertib
Cell 3	*AKT1*	2.386	Cytoplasm	BAY1125976/capivasertib/patasertib/miransertib
Cell 2	*BRD4*	2.241	Nucleus	AZD5153/BI 894999/PLX2853/PLX51107
Cell 2	*CDK1*	2.051	Nucleus	Alvocidib/dinaciclib/milciclib/riviciclib

^a^ Only kinases were listed. ^b^ A maximum of four drugs was listed. ^c^ EGFR and ERBB3 presented gene and protein overexpression. Only the fold change (FC) found in the mRNA analysis is listed.

## Data Availability

The data presented in this study are available on request from the corresponding author.

## Supplementary Materials

The following are available online at https://www.mdpi.com/article/10.3390/cells10040814/s1, Table S1: Genomic alterations shared in primary tumors and their derived PeCa cells, Figure S1: Characteristics of the penile cancer-derived cells; Figure S2: Heatmap of the reverse phase protein array (RPPA) showing the protein expression levels (in alphabetical order); Figure S3: Ideogram of primary tumors (thin lines in pink) and derived cell lines (thin lines in blue) showing the copy number alterations (CytoScan HD, Affymetrix).
