# Supplementary material for: Penile Cancer-Derived Cells Molecularly Characterized as Models to Guide Targeted Therapies
Source: Cells. 2021 Apr 6;10(4):814. doi: 10.3390/cells10040814 (PMC8067406; doi:10.3390/cells10040814)
Supplement: Supplementary file 1 [file cells-10-00814-s001.zip › cells-1141866 supplementary proofreading back.Files - Copy/Suppl.Figures.pdf]

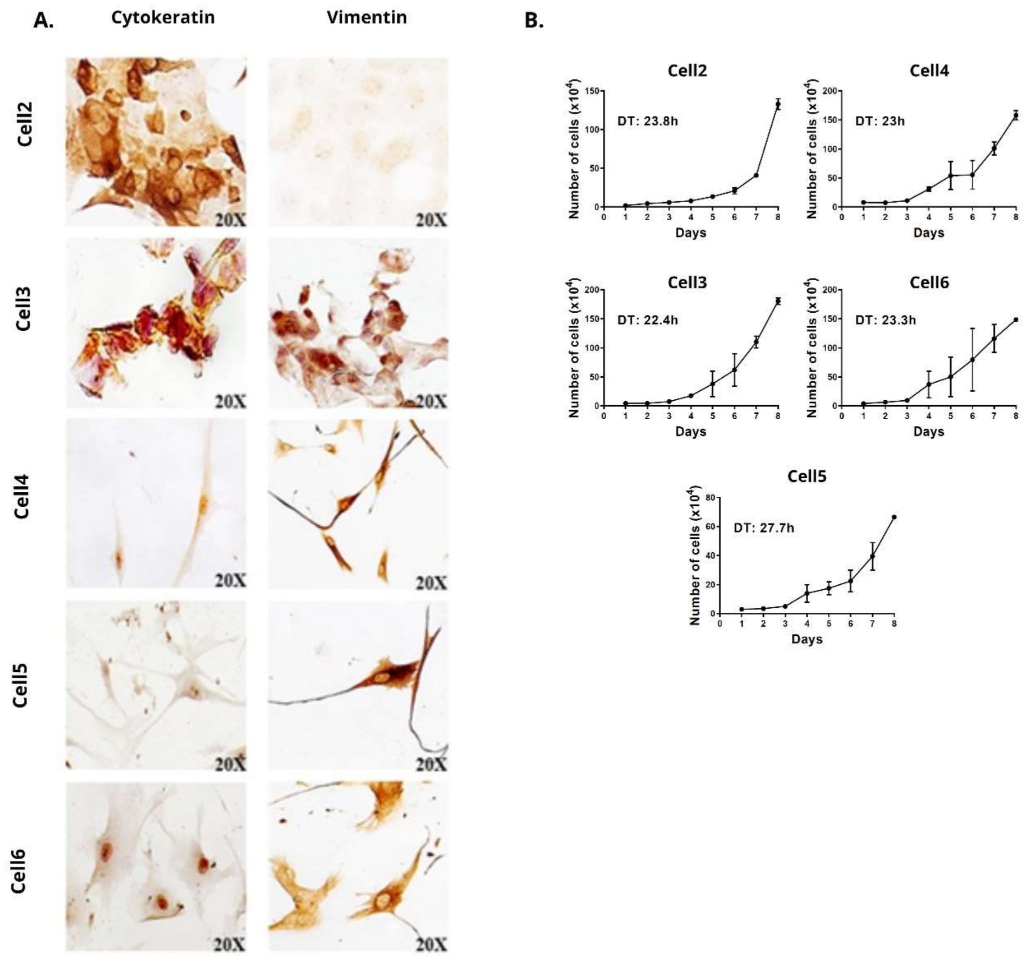

Figure S1. Characteristics of the penile cancer-derived cells (PeCa). (**A**) Representation of Cytokeratin and Vimentin immunocytochemistry staining and (**B**) graphical representation of the doubling time for each of the PeCa-derived cells.

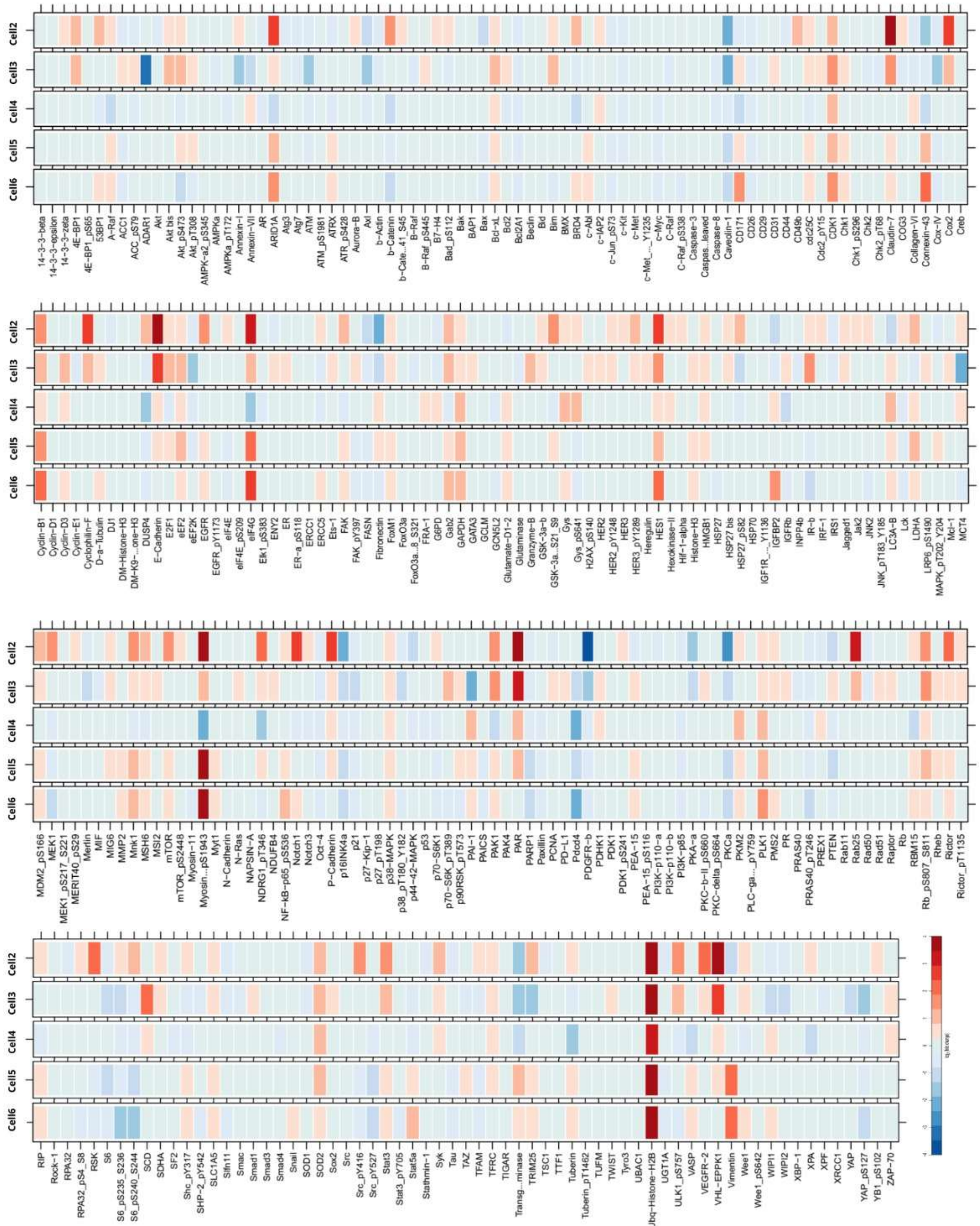

Figure S2. Heatmap of the Reverse Phase Protein Array (RPPA) showing the protein expression levels (in alphabetic order). The protein expression in tumor cells was compared to Cell 1 (reference). Red indicates overexpressed proteins and Blue down-expressed proteins. The figure was generated by RPPApipe tool (Eichner et al., 2014).

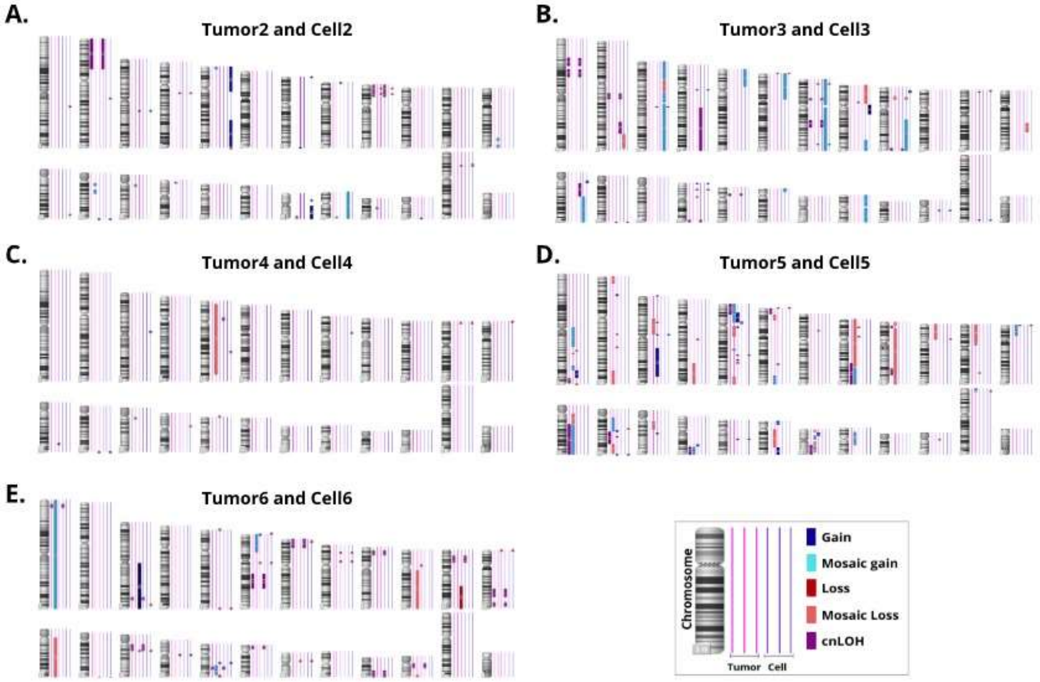

Figure S3. Ideogram of primary tumors (thin lines in pink) and derived cell lines (thin lines in blue) showing the copy number alterations (CytoScan HD, Affymetrix). (**A-E**): A significant number of genomic alterations detected in the primary tumor was retained in the derived cell culture. Regions of copy-neutral loss of heterozygosity (cnLOH) (purple), gains (dark blue), losses (dark red), mosaic gains (light blue), and mosaic losses (light red) are represented. Image adapted from the Affymetrix Chromosome Analysis Suite 3.0 (ChAS) software.
