# Supplementary material for: Penile Cancer-Derived Cells Molecularly Characterized as Models to Guide Targeted Therapies
Source: Cells. 2021 Apr 6;10(4):814. doi: 10.3390/cells10040814 (PMC8067406; doi:10.3390/cells10040814)
Supplement: Supplementary file 1 [file cells-10-00814-s001.zip › cells-1141866 supplementary proofreading back.Files - Copy/Supplementary.tables.pdf]

Table S1. Genomic alterations shared in primary tumors and their derived PeCa cells.

Cell	*Type*	*Chro*	*Start Cell*	*End Cell*	*Size (kbp) Cell*	*Start Tumor*	*End Tumor*	*Size (kbp) Tumor*	*Shared Size (kbp)*
Cell2	Gain	1	155013337	155219172	205.835	154972478	155236611	264.133	205.835
	Gain	3	113898632	113976795	78.163	113898632	113976795	78.163	78.163
	Gain	5	113576	55275365	55161.789	113576	55275365	55161.789	55161.789
	Gain	5	118271056	118573297	302.241	118271055	118573297	302.242	302.241
	Gain	5	119305848	180375290	61069.442	119305848	180375290	61069.442	61069.442
	Gain	5	180418331	180719789	301.458	180418331	180719789	301.458	301.458
	Gain	7	1123531	1566553	443.022	1124365	1562971	438.606	438.606
	Gain	14	106329183	106948691	619.508	106251082	107000205	749.123	619.508
	Gain	19	15779383	15833442	54.059	15780040	15833442	53.402	53.402
	GainMosaic	20	61568	62915555	62853.987	61568	62915555	62853.987	62853.987
	cnLOH	2	15702	68326904	68311.202	15702	68326904	68311.202	68311.202
	cnLOH	9	192128	9261737	9069.609	192128	9317368	9125.24	9069.609
	cnLOH	9	9308229	9785124	476.895	192128	9317368	9125.24	9.139
	cnLOH	9	9793284	21682759	11889.475	9821782	21580763	11758.981	11758.981
	cnLOH	9	22067829	27413212	5345.383	22067829	27413212	5345.383	5345.383
	Loss	2	133582740	133750167	167.427	133576817	133750167	173.35	167.427
	Loss	2	133952092	134007875	55.783	133952092	134007875	55.783	55.783
	Loss	4	69435888	69485827	49.939	69435888	69485827	49.939	49.939
	Loss	5	180378753	180418090	39.337	180378753	180418090	39.337	39.337
	Loss	9	9308229	9785124	476.895	9308229	9785124	476.895	476.895
	Loss	9	21720480	22065883	345.403	21720480	22065883	345.403	345.403
	Loss	13	106229317	106307269	77.952	106229317	106307349	78.032	77.952
	Loss	X	29318639	29347245	28.606	29318639	29347245	28.606	28.606
	Loss	X	32683887	32704020	20.133	32683887	32704020	20.133	20.133
Cell3	Gain	3	93923641	197851986	103928.345	100340054	100442497	102.443	102.443
	Gain	3	100340054	100442497	102.443	100340054	100442497	102.443	102.443
	Gain	4	55102558	55174489	71.931	55103916	55181915	77.999	70.573
	Gain	7	62659911	159119707	96459.796	143425718	144075390	649.672	649.672
	Gain	7	143157525	144123083	965.558	143425718	144075390	649.672	649.672
	Gain	14	106079822	106717343	637.521	106227153	106717343	490.19	490.19
	Gain	16	1002185	1444729	442.544	1052879	1288498	235.619	235.619
	Gain	22	22929813	23262943	333.13	23124497	23262943	138.446	138.446
	Gain	X	148876306	149026550	150.244	148884122	149028748	144.626	142.428
	GainMosaic	5	113576	39317327	39203.751	113576	8894499	8780.923	8780.923
	cnLOH	1	43878711	61457297	17578.586	42956395	61368779	18412.384	17490.068
	cnLOH	1	68692743	85917563	17224.82	68623008	85940105	17317.097	17224.82
	cnLOH	2	115794768	128641375	12846.607	115294660	128856553	13561.893	12846.607
	cnLOH	7	43360	58025873	57982.513	42001908	47626513	5624.605	5624.605

	cnLOH	7	42030332	47544242	5513.91	42001908	47626513	5624.605	5513.91
	cnLOH	7	62659911	159119707	96459.796	90820629	107313947	16493.318	16493.318
	cnLOH	7	90924002	107313727	16389.725	90820629	107313947	16493.318	16389.725
	cnLOH	9	72007158	141020389	69013.231	133621503	141025328	7403.825	7398.886
	cnLOH	9	135205005	141025328	5820.323	133621503	141025328	7403.825	5820.323
	cnLOH	16	84052661	90163275	6110.614	83896234	90163275	6267.041	6110.614
	cnLOH	17	14772900	21076194	6303.294	14767823	21431579	6663.756	6303.294
	Loss	6	156974	57898477	57741.503	254253	314949	60.696	60.696
	Loss	6	253196	294826	41.63	254253	314949	60.696	40.573
	Loss	7	43360	58025873	57982.513	11976266	12098696	122.43	122.43
	Loss	7	11976266	12098696	122.43	11976266	12098696	122.43	122.43
	Loss	8	158048	42360978	42202.93	39247097	39386952	139.855	139.855
	Loss	16	16248154	16257822	9.668	16248154	16258276	10.122	9.668
	LossMosaic	9	21565673	27885795	6320.122	19740519	28296201	8555.682	6320.122
	Gain	14	106072250	106728149	655.899	106401470	107081338	679.868	326.679
Cell4	cnLOH	5	39434096	45181612	5747.516	8501227	166435481	157934.254	5747.516
	Loss	5	115586676	115625244	38.568	8501227	166435481	157934.254	38.568
	Loss	11	5788078	5809230	21.152	5754065	5868830	114.765	21.152
	Gain	3	85614312	85723256	108.944	85619047	85731498	112.451	104.209
	Gain	5	39434096	45181612	5747.516	20432744	42559770	22127.026	3125.674
	Gain	14	106072250	106728149	655.899	106281952	107093267	811.315	446.197
	Gain	17	44212823	44784639	571.816	44212823	44789621	576.798	571.816
	GainMosaic	5	39434096	45181612	5747.516	113576	42903265	42789.689	3469.169
	GainMosaic	12	2235940	2257908	21.968	173786	25391893	25218.107	21.968
Cell5	GainMosaic	14	48227023	48277484	50.461	20511672	53979814	33468.142	50.461
	GainMosaic	X	7684969	7690004	5.035	168546	9346320	9177.774	5.035
	Loss	5	115586676	115625244	38.568	115586765	115625174	38.409	38.409
	Loss	6	257064	302236	45.172	254253	294826	40.573	37.762
	Loss	12	2235940	2257908	21.968	2235940	2257908	21.968	21.968
	LossMosaic	3	85614312	85723256	108.944	49650025	85978802	36328.777	108.944
	LossMosaic	10	34626271	34670662	44.391	100026	35377725	35277.699	44.391
	Gain	3	171650016	177896902	6246.886	93519464	197851986	104332.522	6246.886
	Gain	3	189363665	189371964	8.299	93519464	197851986	104332.522	8.299
	Gain	14	106251082	106728149	477.067	106206397	106728149	521.752	477.067
	Gain	16	34466678	34755816	289.138	34471297	34755816	284.519	284.519
	Gain	17	44187491	44292676	105.185	44190670	44785015	594.345	102.006
Cell6	GainMosaic	1	9559062	18903819	9344.757	849466	249224684	248375.218	9344.757
	GainMosaic	6	257064	294826	37.762	156974	41709481	41552.507	37.762
	GainMosaic	17	55078940	62659557	7580.617	51032211	74219909	23187.698	7580.617
	cnLOH	1	9559062	18903819	9344.757	9559062	18903819	9344.757	9344.757
	cnLOH	3	171650016	177896902	6246.886	171650016	177969526	6319.51	6246.886
	cnLOH	6	91072976	124601694	33528.718	91072976	124601694	33528.718	33528.718
	cnLOH	7	44166	20373272	20329.106	44166	20366528	20322.362	20322.362

cnLOH	7	6838710	6955038	116.328	44166	20366528	20322.362	116.328
cnLOH	9	7660298	19156258	11495.96	7684316	19156258	11471.942	11471.942
cnLOH	11	13768734	29214412	15445.678	13768734	29214412	15445.678	15445.678
cnLOH	12	87920495	106673815	18753.32	87920495	106673815	18753.32	18753.32
cnLOH	12	118139858	128564354	10424.496	118139858	128564354	10424.496	10424.496
cnLOH	15	22752398	39159075	16406.677	22752398	39159075	16406.677	16406.677
cnLOH	17	55078940	62659557	7580.617	55078940	62659557	7580.617	7580.617
cnLOH	18	3001285	10314711	7313.426	3001285	10314711	7313.426	7313.426
cnLOH	21	30741453	48084820	17343.367	30741453	48084820	17343.367	17343.367
cnLOH	22	16877134	28382390	11505.256	16877134	29146503	12269.369	11505.256
Loss	5	180378874	180430789	51.915	180378753	180430789	52.036	51.915
Loss	6	67004972	67051189	46.217	67004972	67051189	46.217	46.217
Loss	8	18851526	18866227	14.701	18851526	18866135	14.609	14.609
Loss	8	39247097	39386952	139.855	39247097	39384337	137.24	137.24
Loss	12	2245848	2257908	12.06	2235940	2258395	22.455	12.06
Loss	15	39669518	39762376	92.858	39669518	39762376	92.858	92.858
Loss	19	20598429	20720705	122.276	20596105	20720705	124.6	122.276

cnLOH: copy-neutral loss of heterozygosis; kbp: kilo-base pair.
